# Transarterial Embolization in Neonatal Kasabach–Merritt Syndrome

**DOI:** 10.3389/fped.2021.788120

**Published:** 2021-12-01

**Authors:** Yinghao Wang, Song Wang, Lili Wang, Shaohua Bi, Jian Zhang, Ping Zha, Liying Dai

**Affiliations:** ^1^Department of Neonatology, Anhui Provincial Children's Hospital, Anhui Medical University, Hefei, China; ^2^Department of Radiology, Anhui Provincial Children's Hospital, Anhui Medical University, Hefei, China

**Keywords:** neonate, transarterial embolization (TAE), Kasabach-Merrit syndrome, bleomycin, hemangioma

## Abstract

**Background:** Kasabach–Merritt syndrome (KMS) is characterized by large hemangiomas and persistent thrombocytopenia, which may result in visceral hemorrhage and disseminated intravascular coagulation. This study aimed to evaluate the value of transarterial embolization (TAE) in neonatal KMS patients.

**Patients and Methods:** The clinical course of 11 neonates with KMS who underwent TAE in the Department of Neonatology, Anhui Provincal Children's Hospital, Anhui Medical University, China, were reviewed retrospectively.

**Results:** Eleven neonates with KMS (nine male and two female) were admitted to our hospital between the age of 1 h and 6 days. All were born with progressively enlarged hemangiomas and persistent thrombocytopenia. The largest lesion had its maximum size reached at 15 × 8 × 8 cm. Eight patients had cutaneous hemangiomas (1 right face, one oropharynx, one left upper arm, two back, one left lumbar, one right lower leg, and one right thigh), and three patients had liver hemangiomas. All 11 patients underwent TAE. Nine patients underwent two TAEs, and two patients underwent only one embolization procedure. They all obtained >80% devascularization of their lesions without a major complication. The platelet count increased at 2–5 days after treatment and reached normal count and coagulation profile at 18–28 days after the TAE.

**Conclusions:** TAE is a safe and effective alternative therapy for neonatal KMS patients.

## Introduction

The Kasabach–Merritt syndrome (KMS), also known as giant hemangioma–thrombocytopenia syndrome, was first reported by Kasabach and Merritt in 1940 (1). In 1997, Sarkar revised the KMS to Kasabach-Merritt phenomenon (2). Approximately 80% of all KMS occur within 1 year of birth, with fatality rates ranging between 10 and 37% (3). It is characterized by large hemangiomas, persistent thrombocytopenia, and bleeding tendency, causing joint and limb dysfunction and potentially leading to serious visceral bleeding and disseminated intravascular coagulation (DIC) (4). Glucocorticoids, sirolimus, or propranolol is most commonly used to treat KMS (5). However, the long treatment course and high rate of side effects prompt us into seeking other therapeutic alternatives (6–8). Transarterial embolization (TAE) has been introduced to manage KMS for about 20 years, with decent results in experienced hands (9, 10). We have previously analyzed the clinical features of 16 neonates with KMS and found that TAE had a good effect in some neonates (11). In this report, we reviewed our own experience of KMS treated with TAE.

## Materials and Methods

This study protocol was approved by the ethics committee of Anhui Provincial Children's Hospital, Anhui Medical University, and informed consent was obtained from the parents. We conducted a retrospective chart view of neonates diagnosed with KMS and who were admitted between January 2016 and June 2021. Eleven cases were identified with (1) platelet count <50 × 10^9^/L and (2) treated by TAE. The sex, age of onset, location and volume of the lesion, laboratory results, image findings, treatment methods, and outcome were retrieved from the electronic medical records.

### Treatment Methods

The medical therapy included glucocorticoids (prednisolone, 2 mg/kg/day; methylprednisolone, 2 mg/kg/day; or dexamethasone, 1 mg/kg/day), propranolol (1.5–2 mg/kg/12 h), and sirolimus (0.025 mg/kg/day).

The femoral artery was cannulated under general anesthesia using the Seldinger technique. A 4-F femoral artery sheath was placed to establish the arterial access, followed by heparinization. Selective arteriography of the lesion using a Cobra-2 catheter (Terumo Corporation, Tokyo, Japan) was performed to identify the feeding artery of the hemangioma. A 2.7-F Progreat catheter (Terumo Corporation, Tokyo, Japan) was then used to catheterize the main feeding artery directly. A mixture of bleomycin (8–10 mg/m^2^), lipiodol (2 ml), dexamethasone (1 mg), and contrast agents, was injected into the main feeding artery until lipiodol was evenly distributed in the lesion. Polyvinyl alcohol (PVA) will be given directly to the feeding artery if the blood flow velocity in the feeding artery remained high. The size of the PVA particles was selected according to the filling time of the hemangioma. PVA particles, 300–500 μm in diameter or 500–700 μm in diameter, were used when the filling time was 2 to 3 s or >3 seconds, respectively. No PVA particles were given with filling time of <2 s. Patients with active bleeding or platelet count <20 × 10^9^/L received platelet infusions, and patients with DIC were treated with cryoprecipitate or prothrombin complex before and after the TAE.

### Therapeutic Assessment

The procedure was considered technically successful if >80% of the tumor vasculature was occluded (10). The hemangioma volume, platelet count, and coagulation function were measured 4 weeks after TAE. The efficacy of TAE was assessed based on two factors: (1) the reduction in tumor volume was estimated *via* ultrasonography using the following formula: width × depth × height × π/6 (width × depth × height × 0.52), based on the shape of the lesion (12), and (2) the platelet count was increased to >50 × 10^9^/L, and the coagulation function returned to normal.

### Data Analysis and Statistics

SPSS version 20.0 (IBM Corporation, Chicago, Illinois) for Windows (Microsoft Corporation, Redmond, Washington) was used for statistical analyses. All statistics were tested to be of normal distribution. Data are shown as mean ± standard deviation (m ± SD), median (interquartile range) [M (p25, P75)], or relative percentages. A paired *t*-test was used for continuous data. Statistical significance was defined as *p* <0.05.

## Results

### Characteristics of the Patients

The clinical data of nine male and two female patients are shown in Table 1. Their age of admission ranged from 1 h to 6 days. They were 10 term infants and one preterm infant, with gestational age of 38.4 ± 1.1 weeks and birth weight of 3,267 ± 341 g.

**Table 1:** Clinical characteristics upon admission.

**Case**	**Sex**	**Age**	**Location**	**Lesion volume**	**Platelet count**	**PT**	**APTT**	**Fibrinogen**	**D-dimer**
		**(h)**			**(×10^**9**^/L)**	**(s)**	**(s)**	**(g/L)**	**(mg/L)**
1	M	16	Back	8.4 × 7.4 × 5.3	8.0	15.5	51.2	0.6	31.0
2	F	24	Right lower leg	6.9 × 4.7 × 2.3	31.0	12.2	36.2	0.9	97.5
3	M	1	Left upper arm	15.0 × 8.0 × 8.0	20.0	120.0	86.1	0.3	80.0
4	M	48	Oropharynx	5.1 × 4.9 × 3.8	13.0	120.0	69.6	0.4	35.2
5	M	72	Liver	10.2 × 6.8 × 5.6	30.0	14.8	64.6	1.4	28.0
6	M	100	Liver	7.0 × 4.9 × 7.0	25.0	120.0	180.0	0.4	23.0
7	M	6	Liver	6.0 × 4.0 × 4.0	4.0	120.0	180.0	0.4	37.0
8	F	144	Right face	7.0 × 6.0 × 3.0	39.0	15.8	42.0	0.3	44.0
9	M	72	Right thigh	8.0 × 4.0 × 4.0	10.0	20.5	50.6	1.3	80.0
10	M	48	Left lumbar	5.4 × 4.0 × 2.6	20.0	14.0	39.4	1.0	28.0
11	M	10	Back	10.5 × 10.2 × 6.4	4.0	19.3	35.4	0.4	40.2

*M, male; F, female; PT, prothrombin time; APTT, activated partial thromboplastin time*.

### Clinical Presentation

**Figure 1:**
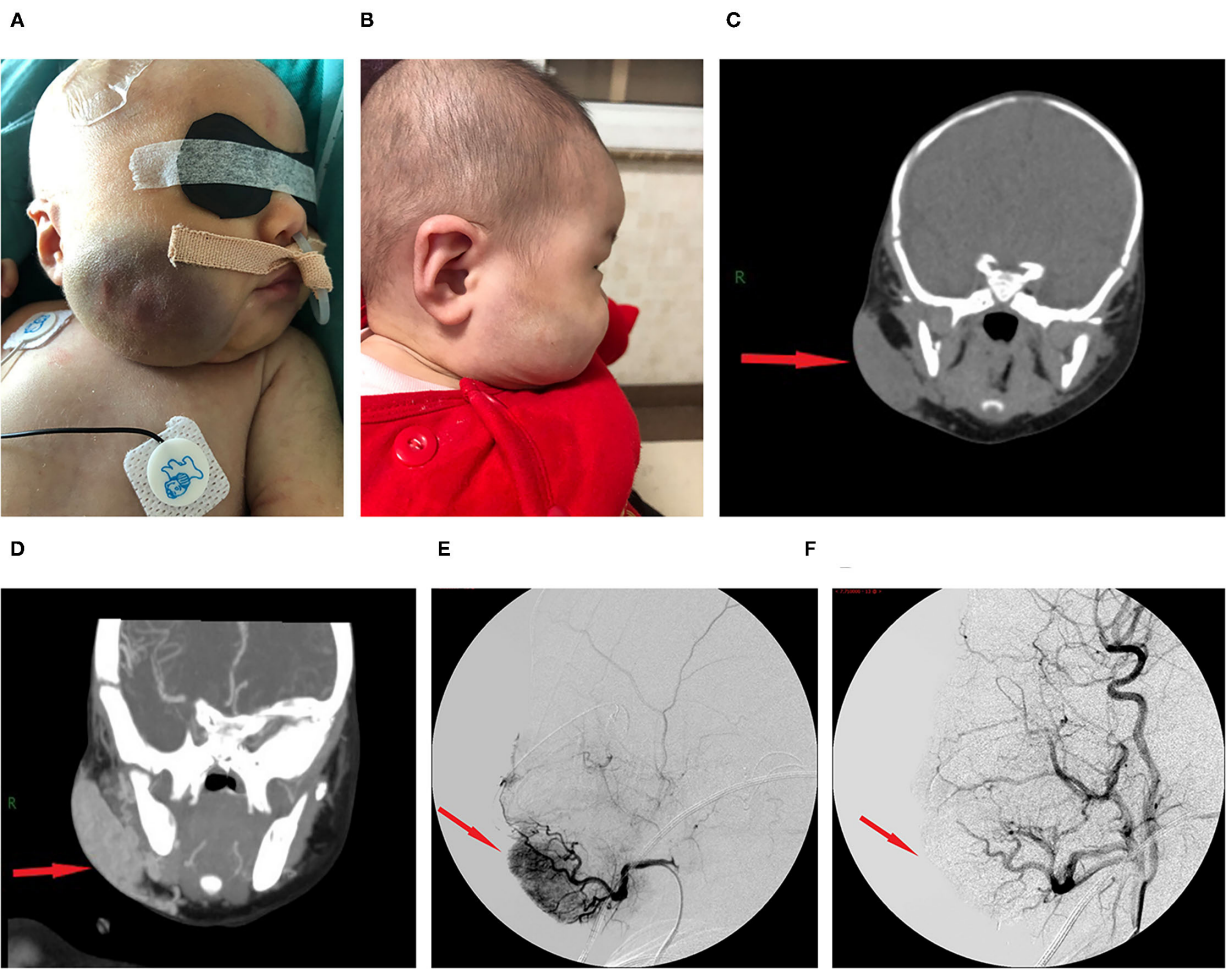
Images from case 8 before and after TAE. **(A)** A large tumor that measured 7.0 × 6.0 × 3.0 cm was seen on the right face. The skin of the tumor was different from the surrounding area, with purple or dark color, obvious swelling, increased tension, slightly elevated but with ill-defined margins, and induration. **(B)** At 5 months after TAE, the tumor almost involuted completely. **(C)** CT showed that the lesion was a large soft tissue mass. **(D)** Contrast-enhanced CT showed obvious arterial phase enhancement inside the mass. **(E)** CTA showed an abundant tumor blood supply before embolization. **(F)** After TAE, more than 80% of the tumor-feeding arteries were embolized. CTA, computed tomography–angiography; TAE, transarterial embolization. The red arrow indicates the lesion.

**Figure 2:**
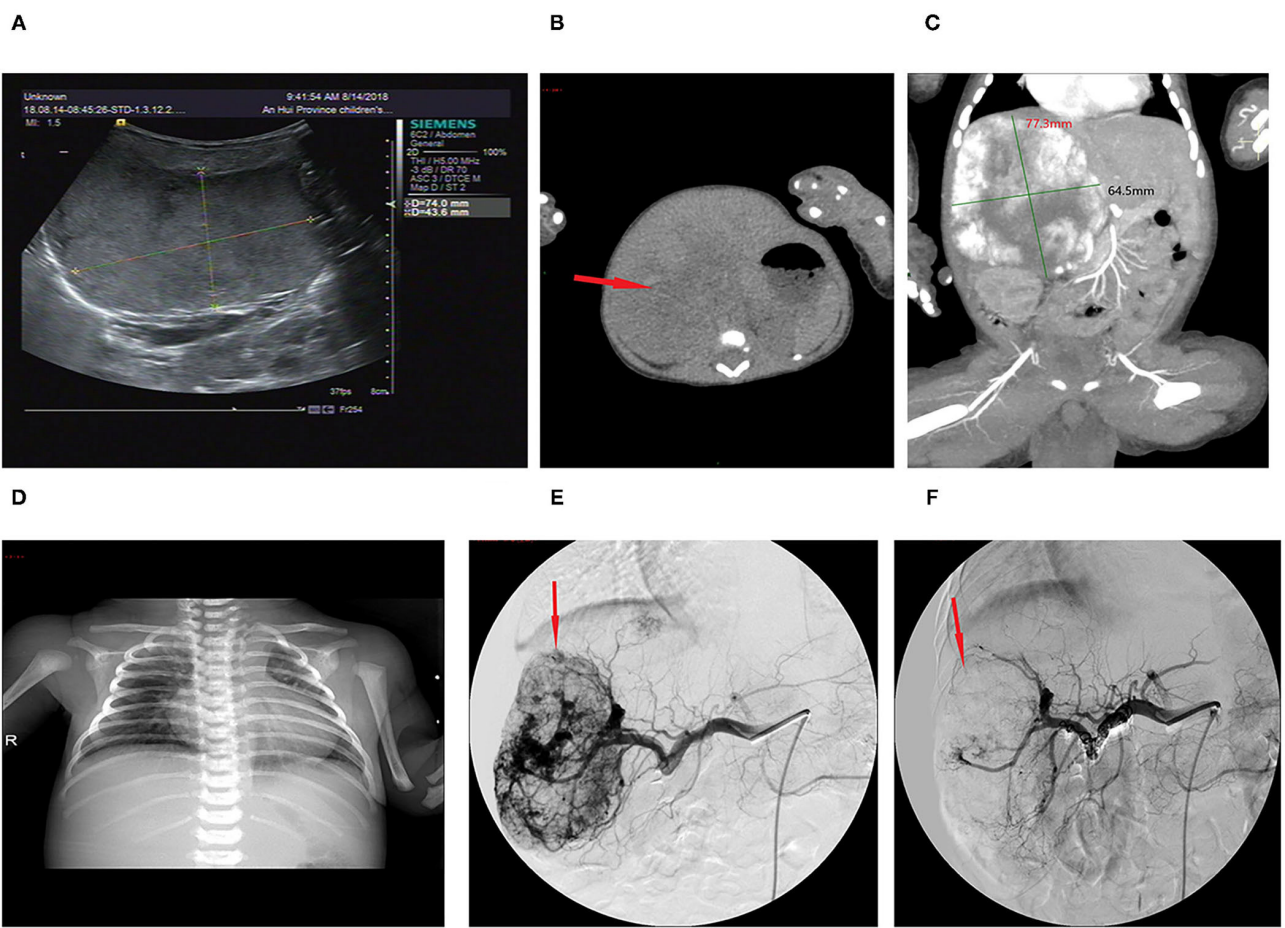
Imaging of the hepatic hemangioma for case 6. **(A)** Ultrasonography showed a hyperechoic solid lesion in the liver. **(B)** CT showed that the lesion was a large soft tissue mass. **(C)** Contrast-enhanced CT showed obvious arterial phase enhancement inside the mass. **(D)** CXR showed enlargement of the heart shadow. **(E)** CTA showed an abundant tumor blood supply before embolization. **(F)** After TAE, more than 80% of the tumor-feeding arteries were embolized. TAE, transarterial embolization; CTA, computed tomography–angiography; CXR, chest X-ray. The red arrow indicates the lesion.

The lesion size all increased with time, with the largest size reaching 15.0 × 8.0 × 8.0 cm; eight had cutaneous hemangiomas (one right face, one oropharynx, one left upper arm, two back, one left lumbar, one right lower leg, and one right thigh), and three had liver hemangiomas. The cutaneous hemangiomas presented as purple to dark red lesions. The skin of the tumor was different from the surrounding area, with obvious swelling, tension, slightly elevated but ill-defined margins, and induration (see Figure 1A). CT showed that the lesion was a large soft tissue mass, contrast-enhanced CT showed obvious arterial phase enhancement inside the mass, and computed tomography–angiography (CTA) showed an abundant tumor blood supply (see Figures 1C–E). In the three patients with liver hemangiomas, ultrasonography showed a hyperechoic solid lesion in liver. The CT showed that the lesion was a large soft tissue mass. Contrast-enhanced CT showed obvious arterial phase enhancement inside the mass. CTA showed an abundant tumor blood supply, and chest X-ray showed enlargement of the heart shadow after admission (see Figures 2A–E). One patient with enlarged cardiac silhouette soon developed heart failure. The condition of all 11 patients was complicated with diffuse ecchymosis and poor response to medical treatment.

### Laboratory Findings

Thrombocytopenia and coagulation dysfunction were observed in all patients. Laboratory examinations showed the platelet count of 20.0 (8.0, 30.0) × 10^9^/L. The prothrombin time (PT) was 19.3 s (14.8, 120.0), with a prolonged PT in six patients. The activated partial thromboplastin time (APTT) was 51.2 s (39.4, 86.1), with a prolonged APTT in six patients. The fibrinogen level was 0.4 g/L (0.4, 1.0), with a low fibrinogen level in all 11 patients. The D-dimer level was 37.0 mg/L (28.0, 80.0), and all values were abnormally increased.

### Treatment and Assessment

The initial therapy was supportive after admission. Five patients with a platelet count of <20 × 10^9^/L received apheresis platelet transfusion. Seven patients with a fibrinogen level of <0.6 g/L received cryoprecipitate infusions. All 11 patients received glucocorticoid treatment prior to TAE. After 3–10 days of treatment, the platelet count remained low, and the hemangioma volume failed to decrease, with hemangiomas that kept on growing in size for five cases. Four patients continued to be treated with propranolol, and two patients continued to be treated with sirolimus and maintained the serum levels between 8 and 15 ng/ml, but their condition remained unimproved. All 11 patients then underwent TAE. Nine patients underwent two embolizations, and two patients underwent only one embolization. Embolization with >80% tumor devascularization was technically successful in all 11 patients without a major complication (see Figures 1F, 2F). The platelet count started to increase 2–5 days after treatment, reaching a normal value at 18–28 days later, by which time the coagulation function had also returned to normal. The platelet count increased from 20.0 × 10^9^/L (8.0, 30.0) before treatment to 255.0×10^9^/L (200.0, 280.0) weeks after treatment (*p* <0.001, see Table 2). The hemangioma size was 50.2 ± 5.3% smaller 4 weeks after TAE, and the maximal diameter significantly decreased from 8.1 ± 2.9 cm before treatment to 6.3 ± 2.8 cm (*p* < 0.001, see Table 2). An almost complete involution of the tumor was observed in case 8 at the 5-month follow-up visit (see Figure 1B).

**Table 2:** Hemangioma volume and platelet count before and after TAE (mean ± SD, *n* = 6).

**Time**	**Hemangioma**	**Platelet count (× 10^**9**^/L)**
	**maximal diameter (cm)**	
Pre-TAE	8.1 ± 2.9	20.0 (8.0, 30.0)
Post-TAE	6.3 ± 2.8	255.0 (200.0, 280.0)
*p*-value	<0.001	<0.001

*TAE, transarterial embolization*.

## Discussion

KMS is a rare condition that results in vascular tumors in infants and is associated with Kaposi hemangioendothelioma and tufted hemangioma in over 70% of cases (5). KMS occurs most frequently in neonates and infants, with approximately 80% of cases occurring within 1 year after birth (3). All our cases were diagnosed within 1 week of life. KMS is characterized by life-threatening thrombocytopenia. It is believed that thrombocytopenia is due to trapping by the hemangioma and activation and aggregation of platelets and reduced fibrinogen levels (5, 13). High cardiac output congestive heart failure, which can significantly complicate the clinical management, occurs in one of our cases.

Medical treatment is always the initial management for KMS (5). Our patients were first treated with glucocorticoids, propranolol, or sirolimus, but the effect was poor. The severity of KMS is proportional to the hemangioma volume, with the greater the volume the more platelet consumption. Liver and retroperitoneal hemangiomas tend to have serious complications, such as heart failure secondary to increased venous return or high cardiac preload that can lead to congestive heart failure (14), and aggressive intervention is warranted. The surgical resection of KMS is not recommended due to the technical difficulty and high risk of intraoperative bleeding, postoperative infection, and extensive scarring, so surgical resection is not the first choice for liver hemangiomas (15) and also not applicable to head and face hemangiomas (12). TAE has been used in the treatment of vascular tumors for many years, with the advantages of minimal trauma, safety and reliability, and quick postoperative recovery by reducing the blood supply to the hemangioma, attenuating heart failure, and effectively promoting the regression of the hemangioma (9, 10, 12).

KMS patients with large tumors or tumors located in the liver and have severe thrombocytopenia can undergo TAE, which was first reported by Yamamoto et al. (16). In our hospital, TAE, using bleomycin and PVA as sclerosing agents, has been routinely used for treating hemangiomas (12). Bleomycin shrinks hemangioma through multiple mechanisms, such as suppressing the metabolism of tumor cells by directly degrading DNA, which then accelerates tumor cell necrosis (17), destroying endothelial cells by the formation of microthrombi in hemangioma sinuses, leading to atrophy and fibrosis of the hemangioma (18) and obliterating the vascular lumen by activating the mTOR pathway and inducing non-specific inflammatory processes around the hemangioma (19). Embolization with PVA exerts a permanent embolic effect by adhering to the vascular wall and inducing an inflammatory cascade with angionecrosis (20). All our 11 patients underwent TAE with >80% tumor devascularization and without major complications. The size of hemangioma decreased approximately 50% at 4 weeks after TAE, with normalization of platelet count and coagulation function. One patient showed a dramatic response with complete disappearance of the tumor in 5 months.

The limitation of embolization is its technical difficulty in young patients. Improper embolization may cause fever, deep tissue necrosis, and scarring and could even be complicated with sepsis. In our study, two patients who underwent one embolization procedure developed a low fever. High doses of bleomycin may damage the alveolar capillaries in neonates and lead to pulmonary fibrosis in severe cases (21), so the dose of a single injection should not exceed 450 mg (22). In sclerosing embolization, attention must be paid to prevent the regurgitation of the embolization agent, which may cause ectopic embolism and other severe unexpected complications. Successfully locating the main feeding artery to the hemangioma is the key for success (12). It is worth mentioning that activated platelets can increase the hemangioma volume by inducing coagulation, resulting in an increased ability of the hemangioma to capture and consume even more platelets, leading, in turn, to an increased risk of bleeding. Since repeated platelet infusions will cause hemangioma to grow, it is recommended that platelet transfusion is suitable only for patients with either platelet count <20 × 10^9^/L with a bleeding tendency or prior to invasive procedures. In our case series, the D-dimer level in four patients was high, up to 97.5 ng/L, while the platelet count decreased before the treatment, but both returned to normal after treatment. The relationship between D-dimer level and the severity of KMS or the platelet count cannot be determined in our cases due to the limited sample size. The long-term outcome of TEA in neonatal KMS remains unknown, and long-term follow-up is important for clinicians.

## Conclusion

KMS is a rapidly progressing vascular lesion complicated with consumptive coagulopathy. Early effective treatment is necessary to prevent associated morbidities. In KMS patients, TAE is a safe and effective alternative therapy and may result in a good prognosis if available. We suggest that patients with KMS can be treated with TAE initially if the hospital has this technology. Our study may be limited by its single-centered, retrospective nature and the small sample size, but we would like to share our successful experience.

## Data Availability Statement

The original contributions presented in the study are included in the article/supplementary material, further inquiries can be directed to the corresponding author/s.

## Ethics Statement

The studies involving human participants were reviewed and approved by Anhui Provincial Children's Hospital, Anhui Medical University. Written informed consent to participate in this study was provided by the participants' legal guardian/next of kin. Written informed consent was obtained from the individual(s), and minor(s)' legal guardian/next of kin, for the publication of any potentially identifiable images or data included in this article.

## Author Contributions

YW and LD conceptualized and designed this study and wrote the paper. SW provided study material. LD and SB contributed to manuscript revision/review. LW was the attending physician. JZ contributed to data analysis and interpretation. PZ contributed to literature research. All authors contributed to the article and approved the submitted version.

## Conflict of Interest

The authors declare that the research was conducted in the absence of any commercial or financial relationships that could be construed as a potential conflict of interest.

## Publisher's Note

All claims expressed in this article are solely those of the authors and do not necessarily represent those of their affiliated organizations, or those of the publisher, the editors and the reviewers. Any product that may be evaluated in this article, or claim that may be made by its manufacturer, is not guaranteed or endorsed by the publisher.
