# Erratum: Transarterial embolization in neonatal Kasabach–Merritt syndrome

**DOI:** 10.3389/fped.2024.1520743

**Published:** 2024-11-26

**Authors:** Frontiers Production Office

**Affiliations:** Frontiers Media SA, Lausanne, Switzerland

**Keywords:** neonate, transarterial embolization (TAE), Kasabach-Merrit syndrome, bleomycin, hemangioma

An Erratum on Transarterial embolization in neonatal Kasabach–Merritt syndrome By Wang Y, Wang S, Wang L, Bi S, Zhang J, Zha P and Dai L (2021). Front. Pediatr. 9:788120. doi: 10.3389/fped.2021.788120

Due to an error, an incorrect affiliation for the handling editor İlgin Özden was published.

The correct affiliation is “Çam & Sakura Hospital, Türkiye”.

The publisher apologizes for this mistake. The original version of this article has been updated.

